# Selective Adsorption of Sr(II) from Aqueous Solution by Na_3_FePO_4_CO_3_: Experimental and DFT Studies

**DOI:** 10.3390/molecules29122908

**Published:** 2024-06-19

**Authors:** Yudong Xie, Xiaowei Wang, Jinfeng Men, Min Zhu, Chengqiang Liang, Hao Ding, Zhihui Du, Ping Bao, Zhilin Hu

**Affiliations:** College of Nuclear Science and Technology, Naval University of Engineering, Wuhan 430033, China; 15971428144@163.com (Y.X.); actwang@sina.com (X.W.);

**Keywords:** strontium, adsorption, Na_3_FePO_4_CO_3_, mechanisms, DFT

## Abstract

The efficient segregation of radioactive nuclides from low-level radioactive liquid waste (LLRW) is paramount for nuclear emergency protocols and waste minimization. Here, we synthesized Na_3_FePO_4_CO_3_ (NFPC) via a one-pot hydrothermal method and applied it for the first time to the selective separation of Sr^2+^ from simulated LLRW. Static adsorption experimental results indicated that the distribution coefficient *K_d_* remained above 5000 mL·g^−1^, even when the concentration of interfering ions was more than 40 times that of Sr^2+^. Furthermore, the removal efficiency of Sr^2+^ showed no significant change within the pH range of 4 to 9. The adsorption of Sr^2+^ fitted the pseudo-second-order kinetic model and the Langmuir isotherm model, with an equilibrium time of 36 min and a maximum adsorption capacity of 99.6 mg·g^−1^. Notably, the adsorption capacity was observed to increment marginally with an elevation in temperature. Characterization analyses and density functional theory (DFT) calculations elucidated the adsorption mechanism, demonstrating that Sr^2+^ initially engaged in an ion exchange reaction with Na^+^. Subsequently, Sr^2+^ coordinated with four oxygen atoms on the NFPC (100) facet, establishing a robust Sr-O bond via orbital hybridization.

## 1. Introduction

Since the mid-20th century, nuclear energy has emerged as a pivotal energy source, harnessed globally for its high energy density that yields substantial electrical power from a compact volume [1]. Yet, the accidental release of radioactive nuclides remains a critical challenge as even minute quantities can inflict significant environmental and health hazards [2,3]. Notably, ^90^Sr, with a half-life (T_1/2_) of approximately 28.8 years, is particularly detrimental due to its high bioactivity and tendency for biological accumulation [4,5]. Like calcium, ^90^Sr is osteotropic, integrating into the skeletal system upon dietary intake [6,7]. It then emits high-energy beta particles that inflict irreversible damage to adjacent tissues. Chronic exposure can precipitate severe pathologies, including bone cancer and cardiovascular diseases, and poses a formidable risk to human health [8]. The hazards of ^90^Sr have garnered widespread attention within the international community. For instance, following the Fukushima nuclear accident in Japan, research into the environmental behavior and biological effects of ^90^Sr became a focal point [9]. Scientists have noted that the migration and transformation processes of ^90^Sr in the marine environment are complex, and their impact on aquatic life should not be overlooked [10]. In addition, the International Atomic Energy Agency (IAEA) has also emphasized the importance of effective monitoring and regulation of ^90^Sr to prevent long-term effects on public health and the environment [11].

Micro-leakages of ^90^Sr are often blended with other effluents, yielding large volumes of low-level radioactive waste (LLRW) with a low radioactive and non-radioactive nuclides ratio [12]. Conventional LLRW treatment strategies, including chemical precipitation [13], solvent extraction [14], oxidation/reduction [15], and membrane [16] technologies, can attenuate radioactivity. However, these techniques face high costs, secondary contamination risks, and operational intricacy. In this context, adsorption technology has become prominent for its selective and efficient removal of radioactive nuclides [17]. Adsorbents that can specifically sequester radioactive nuclides in high-salinity conditions are in demand. These materials are highly sought after, offering operational simplicity, rapid adsorption–desorption kinetics, and robust stability and reusability [18]. Inorganic adsorbents [19] maintain their adsorptive capacity in extreme environments and are frequently utilized for Sr^2+^ removal. Compared to organic counterparts, most inorganic materials exhibit reduced biological toxicity in their raw and by-product forms, offering enhanced environmental compatibility and sustainability [20].

Various inorganic adsorbents have been deployed for Sr^2+^ adsorption, encompassing hydroxyapatite [21], natural and synthetic zeolites [22], clay minerals [23], silicates [24], phosphates [25], titanates [26], metal oxides [27,28], etc. Xu Xia et al. [21] successfully fabricated porous hydroxyapatite from shell powder using a double-diffusion method, resulting in a high specific surface area of 188.5 m^2^·g^−1^. This material achieved a maximum Sr^2+^ removal capacity of 45.36 mg·g^−1^ at 313.15 K and a removal efficiency of 98.94%, with efficiency above 70% after five cycles. Aurélie Merceille et al. [29] synthesized sodium titanate and A-type zeolites using hydrothermal and sol–gel methods to remove Sr^2+^ from wastewater. The study noted a marked decline in adsorption performance with increasing concentrations of interfering ions (such as calcium and sodium) and decreasing in solution pH. Andrei Ivanets et al. [30] prepared Zr-Ca-Mg composite phosphates with diverse chemical compositions and evaluated their adsorption efficacy for ^85^Sr and ^90^Sr. The study revealed high adsorption efficiency (*K_d_* > 10^4^ mL·g^−1^) in distilled and low-salinity solutions, which diminished to less than 10^3^ mL·g^−1^ (*K_d_* < 10^3^ mL·g^−1^) in high-salinity environments. Despite their economic viability and radiation resistance, these materials often suffer from poor selectivity and adsorption capacity, significantly reducing efficiency in acidic conditions. Na_3_FePO_4_CO_3_ (NFPC) is a stable inorganic material among the carbonophosphates [31]. In contrast to the majority of polyanionic compounds, carbonophosphates offer distinct advantages, including cost-effective raw materials, a simple synthesis process, and green environmental attributes [31]. The incorporation of weakly acidic carbonate ions into the phosphate matrix substantially bolsters the material’s stability. The stabilization is complemented by an extensive network of robust covalent bonds, which concurrently elevate the material’s surface electronegativity [32]. Furthermore, within the robust framework of carbonate and phosphate groups lies a substantial population of labile sodium ions, which can generate a plethora of ion exchange sites in solution. The high electronegativity and abundance of ion exchange sites endow NFPC with significant potential for the selective adsorption of strontium [33]. Theoretically, the adsorptive capabilities of NFPC are superior to those of the conventional inorganic adsorbents for strontium listed previously. Nevertheless, NFPC has not yet been identified as an adsorbent for the selective uptake of strontium or other metal ions, and its practicality and mechanism for adsorption are still unclear.

Density functional theory (DFT) [34,35,36] is a computational approach predicated on quantum mechanical principles. It is instrumental in delineating materials’ electronic structure and properties by accounting for electron density distribution. DFT is extensively applied across material science, catalysis, condensed matter physics, and biochemistry. DFT surpasses traditional experimental analyses and characterizations (e.g., XPS, FTIR, XRD) by simulating complex environments and dynamic adsorption processes, offering profound insights into the interactions between adsorption sites and molecules at the microscopic level. DFT has been instrumental in studying the coordination chemistry and adsorption behavior of adsorbates, with applications in inorganic materials such as attapulgite [37], boron nitride [38], hematite [39], kaolinite [40], etc. However, no reports exist on NFPC’s adsorption behavior.

This study pioneers the exploration of NFPC as an adsorbent for the selective removal of Sr^2+^, with DFT employed to investigate its adsorption mechanism deeply. The examination began with the impact of adsorption kinetics, isotherms, thermodynamics, and variables such as pH, contact time, temperature, and coexisting ions on Sr^2+^ adsorption. Subsequent characterization using EDS (mapping), FTIR, and XPS analyzed the adsorbent’s elemental, functional group, and chemical state changes before and after Sr^2+^ adsorption. The adsorption configuration was refined using the CP2K software suite, with an electronic density of states, differential charge density, and Bader charge transfer calculations. This comprehensive analysis elucidated the bonding mechanism and adsorption behavior of Sr^2+^ on the NFPC surface, contributing to the advancement of selective adsorption materials for Sr^2+^ sequestration from complex environmental matrices.

## 2. Results and Discussion

### 2.1. Characterization

**Figure 1:**
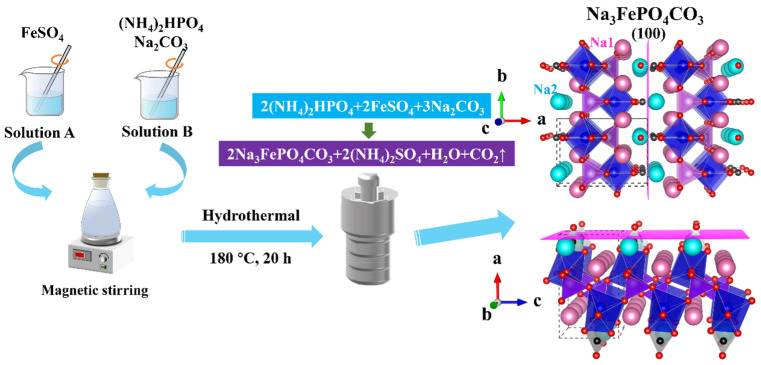
The illustration of the hydrothermal method to prepare NFPC and its crystal structure (Na1, Na2, O, and C atoms are figured in pink, cyan, red, and black, respectively. FeO_6_ and PO_4_ are drawn in blue and Purple, respectively).

**Figure 2:**
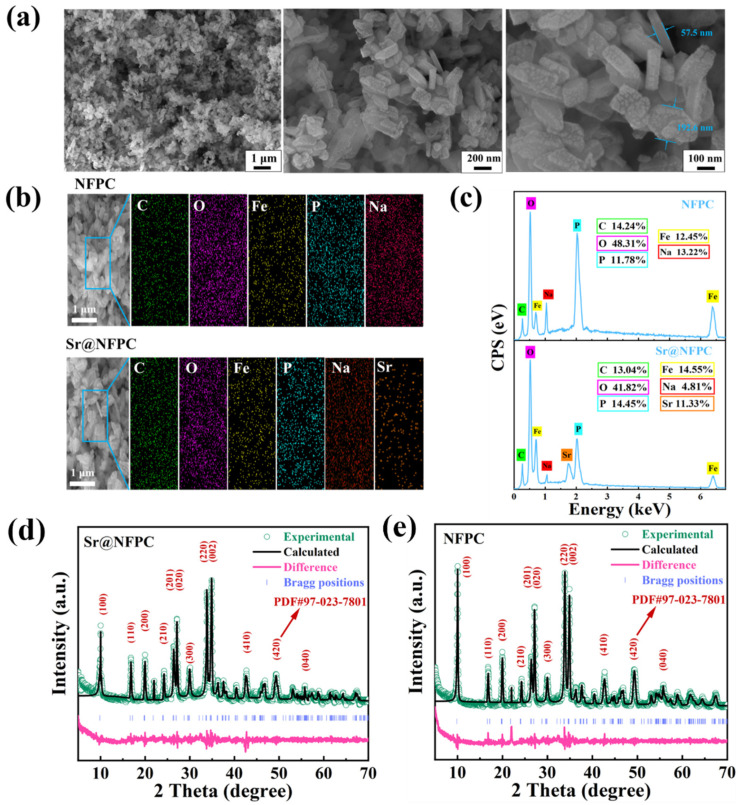
Characterization of NFPC before and after adsorption (Sr@NFPC). (**a**) SEM images of NFPC at different magnifications. EDS results (**b**,**c**) and XRD (**d**,**e**) patterns of NFPC and Sr@NFPC.

The architectural framework of NFPC, classified as sidorenkite, is illustrated in the three-dimensional rendering of Figure 1. The framework is characterized by the interconnection of FeO_6_ octahedra and PO_4_ tetrahedra through corner-sharing, assembling a layered structure. The CO_3_ groups, trigonal and planar, are positioned unilaterally within these layers, sharing two oxygen atoms with adjacent FeO_6_ octahedra. Within the interlayer spaces, sodium atoms are found in two discrete coordination environments: one site (termed Na1) is coordinated with six oxygen atoms, while another (termed Na2) is coordinated with seven [31]. Relative to the Na1 site, the Na2 site features a less robust bond with its coordinating oxygen atoms, endowing it with diminished stability and a heightened susceptibility to dissociation from the underlying framework. This attribute renders the Na2 site a likely candidate for ion-exchange interactions with adsorbate species. As depicted in Figure 2a, microscopic analysis via SEM delineates the NFPC as exhibiting a distinct and uniform block-like morphology, with lateral dimensions varying from 57.5 nm to 192.6 nm. Complementary EDS analysis, presented in Figure 2b, confirms the elemental composition of NFPC as being primarily carbon (C), oxygen (O), iron (Fe), phosphorus (P), and sodium (Na). Following treatment with simulated waste solutions, post-adsorption analysis reveals the presence of Sr^2+^ on the NFPC surface, indicative of effective adsorption. As shown in Figure 2c, elemental mapping facilitates a preliminary deduction of the adsorption mechanism. A notable reduction in the Na content from an initial 13.32% to a subsequent 4.81%, concurrent with an elevation in surface Sr^2+^ content to 11.33%, suggests that Sr^2+^ has been effectively adsorbed onto the NFPC surface. This observation supports a proposed adsorption mechanism involving ion exchange at the Na2 site.

The crystalline integrity of the synthesized NFPC was probed using X-ray diffraction (XRD) analysis both before and after adsorption to ascertain any structural modifications. The findings are detailed in Figure 2d,e. Alignment of the synthesized material with the standard NFPC pattern (PDF#97-023-7801) demonstrated a high degree of correspondence between the prominent diffraction peaks, affirming the successful synthesis [41]. Despite a decrease in the intensity of specific principal peaks after the adsorption process, the peak positions remained unchanged, indicating that the crystalline structure of NFPC was preserved. However, the crystallinity of NFPC was affected, as evidenced by a reduction in overall crystallinity, which can be attributed to the incorporation of adsorbed Sr^2+^.

### 2.2. Adsorption Properties

#### 2.2.1. Effect of pH

**Figure 3:**
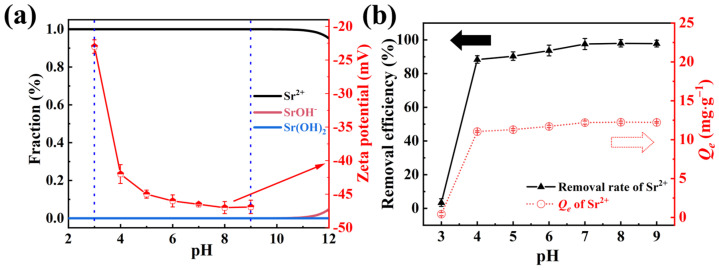
(**a**) The forms of Sr^2+^ and zeta potential at different pH, the red arrow indicates the zeta potential; (**b**) influence of solution pH on the removal efficiency and *Q_e_* (conditions: NFPC dose = 0.4 g·L^−1^; initial Sr^2+^ concentration = 5 mg·L ^−1^; temperature = 303 K; contact time = 120 min), the black and red dashed arrow represent removal efficiency and *Q_e_*, respectively.

The initial solution pH was crucial for the dissociation equilibrium of Sr^2+^ and the surface properties of the adsorbent [42]. To preclude precipitation of Sr^2+^ in solution, the morphologies of Sr^2+^ (initial concentration was 5 mg·L^−1^) were simulated at different pH using Hydra and Medusa software, as depicted in Figure 3a. The findings revealed that Sr^2+^ was the predominant species in solution within the pH range of 1–10, with the onset of Sr(OH)^−^ formation observed at pH values exceeding 10. In engineering applications, the pH range for most low-level waste liquids typically lies between 3–9, where Sr^2+^ remains soluble [43]. Consequently, adsorption experiments were conducted across this pH range. Furthermore, the temperature within the shaking incubator was maintained at 303 K, with an adsorption duration of 2 h.

The effects of solution pH on the Sr^2+^ removal efficiency and adsorption capacity are presented in Figure 3b. At a pH of 3, NFPC exhibited negligible adsorption of Sr^2+^. However, the removal efficiency and adsorption capacity of Sr^2+^ by NFPC increased markedly and asymptotically approached equilibrium with rising pH. This trend can be ascribed to two primary factors: (1) In acidic conditions, the adsorption efficiency of Sr^2+^ is diminished due to competition with protons [44,45]. As the pH increases, deprotonation is enhanced, exposing additional adsorption sites on the adsorbent surface until equilibrium is achieved. (2) The surface charge of the adsorbent is pH-dependent, which directly influences the electrostatic interactions between NFPC and Sr^2+^ [46]. As illustrated by the Zeta potential measurements in Figure 3a, the electronegativity of the adsorbent surface increased significantly with rising pH, mirroring the observed adsorption behavior. Thus, the observed adsorption pattern can be attributed to electrostatic interactions, with the specific adsorption mechanism detailed in Section 2.3.

#### 2.2.2. Effect of Contact Time

**Figure 4:**
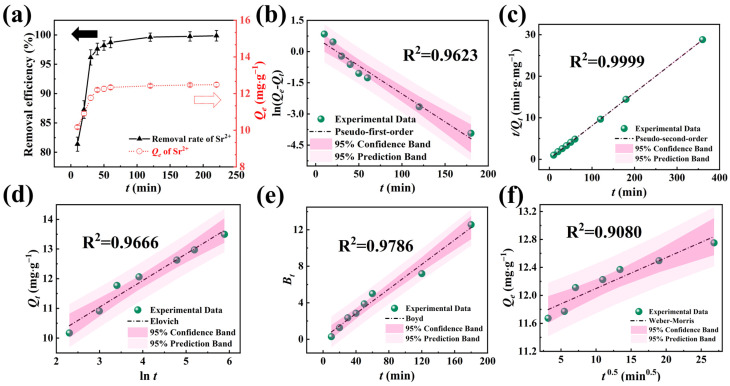
(**d–f**) The influence of contact time on adsorption performance and adsorption kinetics research. (**a**) Influence of contact time on the removal efficiency and *Q_e_* (conditions: NFPC dose = 0.4 g·L^−1^; initial Sr^2+^ concentration = 5 mg·L^−1^; temperature = 303 K; pH = 7), the black and red dashed arrow represent removal efficiency and *Q_e_*, respectively. Pseudo-first-order (**a**), Pseudo-second-order (**b**), Elovich (**c**), Boyd (**a**), and Weber–Morris kinetics results for the adsorption of Sr^2+^.

The influence of contact time (*t*) on the Sr^2+^ removal efficiency and adsorption capacity (*Q_e_*) are delineated in Figure 4a. As time increased, the Sr^2+^ removal efficiency and *Q_e_* gradually rose to equilibrium. Notably, NFPC achieved adsorption equilibrium in approximately 36 min, with a maximum removal efficiency and *Q_e_* recorded at 99.86% and 12.49 mg·g^−1^, respectively.

The pseudo-first order, pseudo-second order, and Elovich models were applied to evaluate the adsorption process better [47,48,49,50,51]. The pseudo-first-order kinetic model posits that the rate-limiting step is diffusion-controlled, while the pseudo-second-order model suggests that chemical interactions predominantly govern the adsorption rate. Conversely, the Elovich model accounts for the heterogeneity of active sites on the adsorbent, leading to variable chemical adsorption energies. The experimental data about the adsorption of Sr^2+^ by NFPC were subjected to fits using these three kinetic models, with the outcomes presented in Figure 4b–d and Table S1. The application of confidence and prediction bands facilitated an assessment of the models’ accuracy and reliability. The pseudo-second-order (PSO) model yielded an equilibrium adsorption capacity of 12.58 mg·g^−1^ for NFPC towards Sr^2+^, closely approximating the experimental value of 12.47 mg·g^−1^, with a negligible error of 0.87%. The PSO model’s fitting line was closest to the experimental data points and exhibited the most compact confidence and prediction bands. Furthermore, the PSO model’s coefficient of determination (R^2^) surpassed 0.99, significantly higher than the other models. Consequently, the PSO model was deemed more adept at characterizing the kinetic behavior of NFPC in the adsorption of Sr^2+^ from aqueous solutions. These findings suggest that the adsorption of Sr^2+^ by NFPC is predominantly mediated by chemical interactions such as ion exchange, electrostatic interactions, and chelation processes.

Because the three aforementioned kinetic models cannot elucidate the diffusion mechanism, we employed the Boyd external diffusion rate model and the Weber–Morris (W-M) internal diffusion rate model to investigate further the factors influencing the adsorption rate. Two types of diffusion predominantly govern adsorption by the adsorbent: film diffusion and intraparticle diffusion. Film diffusion pertains to the migration of the adsorbate from the liquid film surrounding the adsorbent’s surface into its interior. Intraparticle diffusion, on the other hand, describes the subsequent movement of the adsorbate within the adsorbent’s pores or particles. The Boyd model postulates that the primary source of diffusion resistance arises from the film diffusion process. Conversely, the W-M model proposes negligible film diffusion resistance, assuming a constant diffusion coefficient and a random diffusion direction. Figure 4e,f and Table S2 detail the fitting curves and the corresponding parameter values.

The Boyd model demonstrated notably tighter confidence and prediction bands than the Weber–Morris (W-M) model, accompanied by a superior coefficient of R^2^. This observation indicated that the Boyd model has a greater predictive accuracy than the Weber–Morris (W-M) model, rendering it a more suitable framework for characterizing the diffusion mechanism of Sr^2+^ on the surface of NFPC. As detailed in Table S2, the model’s intercept *I_B_* value was recorded at 0.13. The parameter’s absolute value close to zero signifies a strong film diffusion influence on adsorption. Consequently, the adsorption rate of Sr^2+^ was inferred to be predominantly mediated by film diffusion, with the adsorbate being predominantly adsorbed onto the NFPC surface after traversing the liquid film.

#### 2.2.3. Effect of Temperature and Initial Concentration

**Figure 5:**
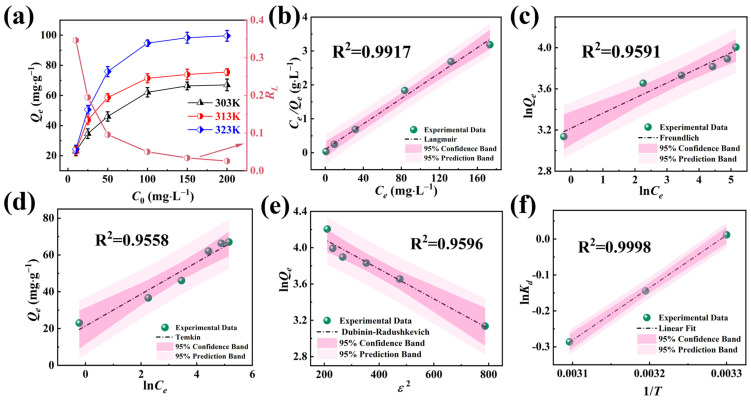
(**a**) Influence of initial Sr^2+^ concentration on *Q_e_* and *R_L_* (conditions: NFPC dose = 0.4 g·L^−1^; pH = 7; contact time = 120 min). Langmuir (**b**), Freundlich (**c**), Temkin (**d**), and Dubinin–Radushkevich (**e**) isotherms result in the adsorption of Sr^2+^. (**f**) The fitting curve between ln*K_d_* and 1/*T*.

The equilibrium adsorption capacities of NFPC across a spectrum of temperatures and initial Sr^2+^ concentrations are presented in Figure 5a. Elevating the initial concentration of Sr^2+^ led to a substantial enhancement in adsorption capacity across all three temperatures studied, with the maximum equilibrium adsorption capacity reaching 67.10 mg·g^−1^ at 303 K.

Initially, the adsorption mechanism of NFPC for Sr^2+^ was probed at a constant temperature, and a range of initial concentrations was found. The experimental data were analyzed using the Langmuir, Freundlich, Dubinin–Radushkevich (D-R), and Temkin models [52,53,54,55,56]. The Langmuir model, which assumes a finite number of uniform adsorption sites on the adsorbent surface with equal adsorption probabilities for target ions, indicates monolayer adsorption without a change in the adsorption layer post-adsorption. The Freundlich model, which posits a heterogeneous surface energy distribution on the adsorbent, is typically applied to multilayer chemical adsorption at lower adsorbate concentrations. The D-R model suggests that adsorption involves the progressive filling of adsorbent pores by the adsorbate, allowing for the differentiation between physical and chemical adsorption mechanisms. The Temkin model hypothesizes a linear relationship between adsorption enthalpy and temperature, with a maximal distribution of uniform bond energies. The fitting curves and pertinent parameters are detailed in Figure 5b–e and Table S3. An exhaustive comparison revealed that the Langmuir model’s confidence and prediction bands were significantly narrower than those of the other three models, with the highest R^2^ of 0.9917. Consequently, the Langmuir model more accurately represents the adsorption process of Sr^2+^ by NFPC, confirming a uniform distribution of adsorption sites and monolayer adsorption. The Langmuir isotherm model predicts a maximum adsorption capacity (*Q_m_*) for Sr^2+^ of 63.48 mg·g^−1^, closely approximating the experimental value of 67.1 mg·g^−1^, bearing a negligible discrepancy of 5.39%. The D-R isotherm model (R^2^ = 0.9596) calculated an average free energy of 15.82 kJ·mol^−1^. The average free energy within the 8 to 16 kJ·mol^−1^ range suggested that the ion exchange mechanism is predominant, aligning with the kinetic model analysis presented in Section 2.2.2. The Freundlich isotherm model (R^2^ = 0.9591), with a constant *n* within the 0–1 range, indicated preferential adsorption of metal ions by NFPC. The variation of the separation factor (*R_L_*) with the initial concentration of Sr^2+^ is depicted in Figure 5a. The *R_L_* value decreased as the initial Sr^2+^ concentration increased, consistently remaining within the 0–1 range. The trend indicated a favorable adsorption of Sr^2+^ by NFPC, and the adsorption process became increasingly advantageous at higher initial concentrations of Sr^2+^.

As illustrated in Figure 5a, the adsorption capacity of NFPC at a constant initial concentration substantially increased with rising temperature, indicative of the endothermic nature of the adsorption process. At an initial Sr^2+^ concentration of 200 mg·L^−1^ and a temperature of 323 K, the equilibrium adsorption capacity peaked at 99.6 mg·g^−1^. To further elucidate the influence of temperature on the adsorption of Sr^2+^ by NFPC, a thermodynamic analysis was conducted to evaluate the adsorption process [57]. The relationship between ln*K_d_* and the inverse temperature (1/T) are presented in Figure 5f. The simulated *K_d_*^0^ values were obtained via Equation (22) in S2.3 [58]. At the same time, the enthalpy change (ΔH) and entropy change (ΔS) were calculated from the slopes and intercepts of the fitted lines in Figure 5f, as detailed in Table S4. The negative values of ΔG and ΔH across the three temperatures confirmed that the adsorption process was a thermodynamically spontaneous exothermic reaction. The negative value of ΔS indicated an enhancement in systemic orderliness throughout the adsorption process, denoting a decrement in molecular mobility in the adsorbed state relative to the solvated state.

#### 2.2.4. Effect of Co-Existing Ions

**Figure 6:**
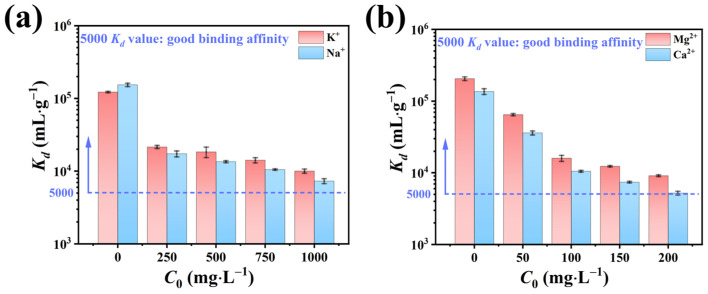
Influence of (**a**) K^+^ and Na^+^ and (**b**) Ca^2+^ and Mg^2+^ ionic strength on the *K_d_* values of Sr^2+^ adsorption (conditions: NFPC dose = 0.4 g·L^−1^; initial Sr^2+^ concentration = 5 mg·L^−1^; temperature = 303 K; pH = 7; contact time = 120 min).

In addition to trace radioactive nuclides such as Sr^2+^, low-level radioactive waste also contains significant quantities of non-radioactive ions, including K^+^, Na^+^, Ca^2+^, and Mg^2+^. To simulate the adsorptive performance of NFPC under realistic environmental conditions, the influence of these varying concentrations on Sr^2+^ adsorption was evaluated. Figure 6 delineates the impact of coexisting ion concentrations on *K_d_* values for Sr^2+^ adsorption, which indicated the adsorbent–adsorbate affinity. A gradual decline in *K_d_* values with increasing coexisting ion concentrations was observed. Notably, even at an elevated concentration of 1000 mg·L^−1^ (200-fold) for K^+^ and Na^+^, the *K_d_* values remained substantially above the threshold of 5000, suggesting a minimal impact on Sr^2+^ adsorption. Conversely, when the Ca^2+^ and Mg^2+^ concentrations were elevated to 200 mg·L^−1^ (40-fold), the *K_d_* values slightly exceeded 5000, implying a more pronounced effect on Sr^2+^ adsorption. This trend could be attributed to the charge density, defined as the ratio of the ionic charge to the hydrated ionic radius [59]. The charge densities for K^+^, Na^+^, Ca^2+^, Mg^2+^, and Sr^2+^ were 0.30 (1/3.31), 0.28 (1/3.58), 0.47 (2/4.28), 0.49 (2/4.12), and 0.49 (2/4.12), respectively. The higher charge densities of Ca^2+^ and Mg^2+^, compared to K^+^ and Na^+^, result in a more significant influence on *K_d_* values. Among these, the charge density of Ca^2+^ matched that of Sr^2+^ and was slightly higher than Mg^2+^, indicating that Ca^2+^ exerts the greatest impact on Sr^2+^ adsorption, followed by Mg^2+^. In conclusion, the influence of coexisting ions on the adsorption of Sr^2+^ is strongly associated with their charge density, with the greatest impact observed when the charge density closely resembles Sr^2+^ [59].

#### 2.2.5. Leachability, Regeneration Property, and Adsorption Performance Comparison

**Figure 7:**
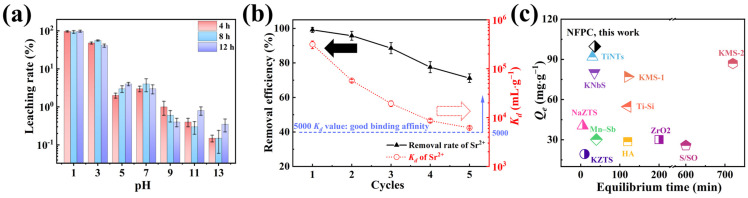
(**a**) Influence of pH and contact time on leaching rate of Sr^2+^ (conditions: Sr@NFPC dose = 0.4 g·L^−1^; temperature = 303 K); (**b**) reusability valuation for Sr^2+^ adsorption of NFPC until five adsorption–desorption cycles (conditions: NFPC dose = 0.4 g·L^−1^; initial Sr^2+^ concentration = 5 mg·L^−1^; temperature = 303 K; pH = 7; contact time = 120 min); (**c**) the values of *Q_e_* and equilibrium time at 303 K in this work are compared to those reported in the literature. HA (Hydroxyapatite) [62]; TiNTs (titanate nanotubes) [63]; KNbS (potassium niobium sulfide) [63]; Mn–Sb (manganese-antimony composite oxide) [59]; Ti-Si (TiO_2_–SiO_2_ mixed gel spheres) [64]; KMS-1 (K_2x_Mn_x_Sn_3-x_S_6_ (x = 0.5−1)) [65]; KMS-2 (K_2x_Mg_x_Sn_3−x_S_6_(x = 0.5−1)) [66]; S/SO (Sb(III)/Sb_2_O_5_) [67]; ZrO_2_ (hydrous zirconium dioxide) [68]; KZTS (K_1.87_ZnSn_1.68_S_5.30_) [69]; NaZTS (Na_5_Zn_3.5_Sn_3.5_S_13_·6H_2_O) [70].

Leachability was a critical parameter for assessing adsorbent materials, with substantial implications for engineering applications [60]. We evaluated the leachability of NFPC under various pH and contact times using a temperature-controlled shaker incubator, with results depicted in Figure 7a. Our findings indicated that the leaching rates of NFPC remain relatively consistent over the 4 h to 12 h interval. Furthermore, strontium exhibited increased leachability under acidic conditions, with the rate escalating as the acidity intensified. At a contact time of 4 h and pH values of 1, 3, and 5, the leaching percentages of strontium were 97%, 48%, and 2%, respectively, correlating with the adsorption trends observed in Figure 3b. This suggested that NFPC, post-strontium adsorption, should be stored in environments that mitigate exposure to strong acids, with conditions above a pH of 5 being more stable. Additionally, this provided insights into the regeneration of NFPC, as after 4 h in contact with a solution at pH 1, a significant desorption of strontium from the NFPC surface was achieved. The reusability of adsorbents was crucial to engineering applications [61]. Consequently, the reusability of NFPC was assessed over five cycles of adsorption/desorption experiments. Figure 7b demonstrates that the removal efficiency and adsorption capacity of NFPC for Sr^2+^ diminishes with increasing cycles. After five cycles, the removal efficiency and *K_d_* values were reduced from their initial values of 99.2% and 3.1 × 10^5^ to 71.2% and 6180, respectively. The decline might stem from incomplete desorption at some sites, leading to decreased adsorption efficacy. Nonetheless, after the fifth desorption experiment, the *K_d_* value remained above 5000 mL·g^−1^, indicating NFPC’s commendable reusability for Sr^2+^ removal from LLRW. In Figure 7c, we juxtaposed the performance of NFPC for Sr^2+^ removal with other inorganic adsorbents reported in the literature. NFPC demonstrated an equivalent or superior adsorption rate and capacity performance compared to previously documented materials.

### 2.3. Adsorption Mechanism

**Figure 8:**
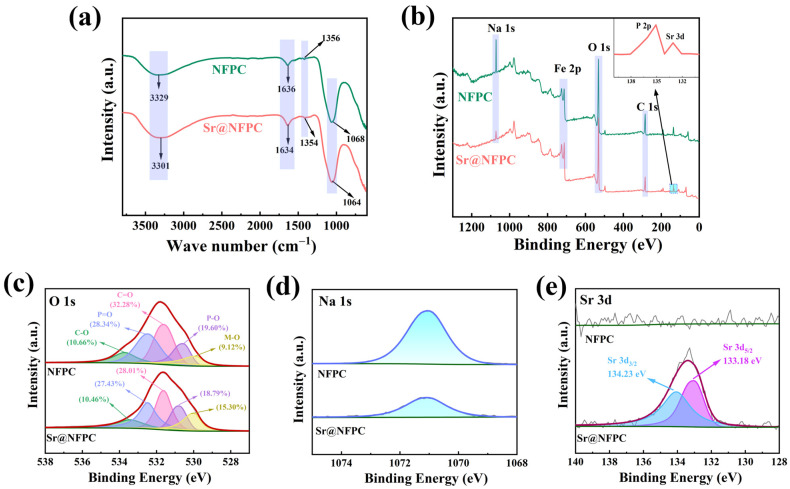
FTIR (**a**) and XPS spectra of NFPC and Sr@NFPC: (**b**) overall spectrum; (**c**) O 1s; (**d**) Na 1s; (**e**) Sr 3d_3/2_ and Sr 3d_5/2_.

To validate the model above fitting results, experimental characterization via FTIR and XPS, in conjunction with DFT calculations, was conducted to explore the adsorption mechanism further. FTIR analysis was performed to ascertain changes in the functional groups of NFPC before and after Sr^2+^ adsorption, as depicted in Figure 8a. The scarcity of FTIR bands for NFPC implied a limited array of functional groups within this inorganic material. The sample’s absorption peak at 3329 cm^−1^, attributable to the stretching vibrations of hydroxyl groups [71], shifted to 3301 cm^−1^ upon adsorption. This shift might result from the forming of hydroxyl groups through the interaction of hydrogen ions with surface oxygen atoms on NFPC. The absorption peaks at 1636 cm^−1^, 1356 cm^−1^, and 1068 cm^−1^ were ascribed to the asymmetric stretching vibrations of C=O, P=O, and C-O, respectively [72,73]. No significant changes in wavelength or intensity were observed post-adsorption, indicating that the NFPC structure remained largely unaltered upon Sr^2+^ adsorption.

XPS is a technique that elucidates a sample’s surface chemical composition and status by analyzing the energy of photoelectrons ejected via X-ray irradiation. It is utilized to investigate the adsorption mechanism further. The XPS analysis of Sr^2+^ coordination on the NFPC surface before and after adsorption is presented in Figure 8. The absence of Sr^2+^ on the NFPC surface before adsorption is confirmed by Figure 8b,e [20]. The O 1s spectrum reveals five distinct oxygen coordination types: carbon–oxygen single bonds (C-O, 10.66%), carbon–oxygen double bonds (C=O, 32.28%), phosphorus–oxygen single bonds (P-O, 19.60%), phosphorus–oxygen double bonds (P=O, 28.34%), and metal–oxygen bonds (M-O, where M signifies metal elements, 9.12%) [74,75,76]. Following NFPC’s interaction with a Sr^2+^-containing solution, Sr^2+^ peaks (133.18 eV and 134.23 eV) in the spectrum signify Sr^2+^ adsorption onto the NFPC surface. According to Figure 8b,d, a noticeable decrease in the intensity of the Na 1s peak is observed after the adsorption reaction, indicating a reduction in the content of some sodium atoms. This decrease implies that ion exchange may occur between Na^+^ and Sr^2+^ during the adsorption process. Subsequent analysis of the O 1s spectrum post-adsorption disclosed alterations in the proportions of the five oxygen-containing bond types to C-O (10.46%), C=O (28.01%), P-O (18.79%), P=O (27.43%), and M-O (15.30%). The proportion of M-O bonds on the NFPC surface notably increased due to ion exchange, where Sr^2+^ replaced sodium ions, forming stronger Sr-O bonds.

**Figure 9:**
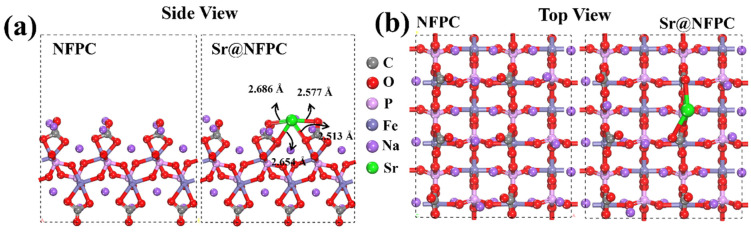
The side view (**a**) and top view (**b**) of the DFT-calculated configuration of NFPC and Sr^2+^ on NFPC (100) surface.

Density functional theory (DFT) was harnessed to investigate the adsorption behavior of Sr^2+^ on NFPC. The underlying periodic structure of NFPC was derived from the Crystallography Open Database (COD). Post-geometric optimization, the most stable configurations before and after the adsorption of Sr^2+^ on the NFPC (100) surface were obtained, as shown in Figure 9. In Figure 9a, Sr^2+^ is observed to interact with four oxygen atoms on the NFPC surface (originating from C=O and C-O/Fe-O) at distances of 2.513 Å, 2.577 Å, 2.654 Å, and 2.686 Å. The total energies of Sr^2+^, NFPC, and Sr@NFPC were calculated to be −30.30 kcal·mol^−1^, −4679.99 kcal·mol^−1^, and −4710.91 kcal·mol^−1^, respectively. The binding energy (*BE*) of Sr^2+^ on the NFPC (100) surface was −380.44 kcal·mol^−1^, underscoring the robust adsorptive effect [37,77].

**Figure 10:**
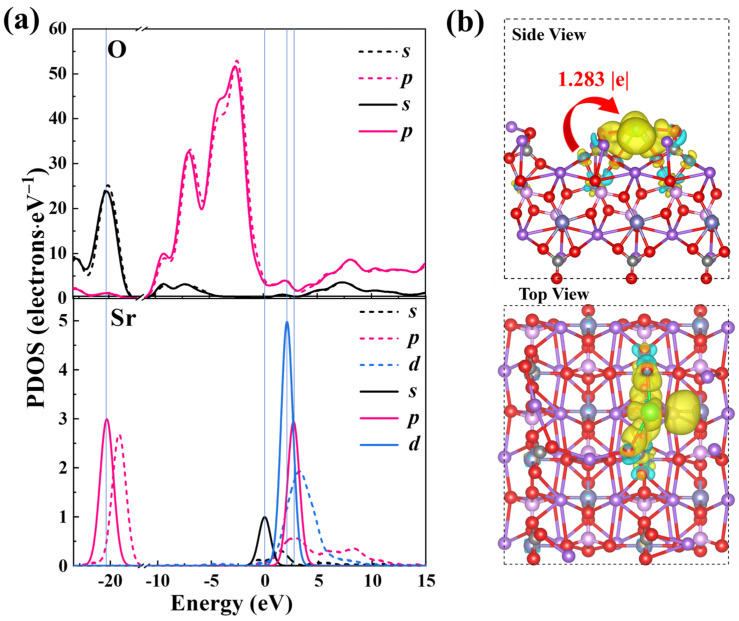
(**a**) PDOS of O and Sr atom before (dotted line) and after (solid line) Sr^2+^ adsorption on NFPC (100) surface; (**b**) the charge density differences and Bader charge transfer of Sr^2+^ adsorption on NFPC (100) surface. Yellow indicates electron accumulation, and light blue indicates depletion.

To attain a profound understanding of NFPC’s selective capture of Sr^2+^, the atomic orbital projected state density (PDOS) and differential charge density before and after Sr^2+^ adsorption were examined, with results depicted in Figure 10. The PDOS analysis is presented in Figure 10a, where the blue vertical lines indicate orbital overlap. After Sr^2+^ adsorption, the O atoms and Sr^2+^ orbitals descended to lower energy states, signifying electron transfer during the adsorption process [78]. Additionally, there was an observed overlap between the *s* and *p* orbitals of O atoms and the *s*, *p*, and *d* orbitals of Sr^2+^. The phenomenon indicated orbital hybridization between NFPC and Sr^2+^, intense charge transfer, and chemical interaction between Sr^2+^ and O atoms [79,80]. The charge difference analysis presented in Figure 10b, coupled with the Bader charge transfer value of 1.283 |e|, confirmed that electrons from O atoms on the NFPC surface were transferred to the Sr^2+^ surface during adsorption. The electron analysis indicated that NFPC facilitates stable Sr^2+^ adsorption through electron transfer from its surface oxygen atoms. In conjunction with the experimental data fitting results detailed in Section 2.2 and the conclusions drawn from XPS analysis, a chemical interaction related to ion exchange between NFPC and Sr^2+^ was revealed.

## 3. Material and Methods

### 3.1. Materials and Reagents

All chemical grades met the experimental requirements and did not require further purification. Ferrous sulfate heptahydrate (FeSO_4_·7H_2_O), diamine hydrogen phosphate ((NH_4_)_2_HPO_4_), and sodium carbonate (Na_2_CO_3_) were purchased from Shanghai Aladdin Biochemical Technology Co., Ltd. (Shanghai, China). Sodium chloride (NaCl), potassium chloride (KCl), magnesium chloride (MgCl_2_), calcium chloride (CaCl_2_), manganese chloride (MnCl_2_), strontium nitrate (Sr(NO_3_)_2_), anhydrous methanol (CH_4_O), and other reagents were purchased from Tianjin Damao Chemical Reagent Co., Ltd. (Tianjin, China). Deionized water (with a conductivity below 5 μS·cm^−1^ at room temperature) was produced by Reverse Osmosis Ultra-Pure Water Equipment from Ningbo Dansibo Environmental Technology Co., Ltd. (Ningbo, Zhejiang, China). The initial pH of each solution was adjusted with dilute HCl or NaOH solution.

### 3.2. Synthesis of NFPC

As shown in Figure 1, Na_3_FePO_4_CO_3_ (NFPC) was prepared via a one-pot hydrothermal synthesis route within a 100 mL polytetrafluoroethylene (PTFE) lined autoclave enclosed by a steel shell [31]. Initially, 8 mmol of iron sulfate heptahydrate (FeSO_4_·7H_2_O) was dissolved in 20 mL of deionized water to achieve a homogeneous solution (solution A). Concurrently, a separate solution was prepared by dissolving a total of 8 mmol of diammonium hydrogen phosphate (NH_4_)_2_HPO_4_ and 0.075 mol of sodium carbonate (Na_2_CO_3_) in 40 mL of deionized water, resulting in solution B. Subsequently, solution A was rapidly introduced to solution B under vigorous magnetic stirring to form a mixed slurry. The slurry was then poured into the PTFE-lined reactor and subjected to hydrothermal treatment at a temperature of 180 °C for 20 h. Upon completion of the reaction period, the reactor was allowed to cool naturally to ambient temperature. The resultant slurry was centrifugally separated, followed by successive washing with deionized water and methanol to purify the solid product. The solid samples were dried under vacuum at 40 °C for 12 h to obtain the NFPC product.

### 3.3. Characterization

Scanning electron microscopy (SEM; Hitachi-3400N, Hitachi Production Co., Ltd.; Tokyo, Japan) coupled with energy-dispersive X-ray spectroscopy (EDS; INCA ENERGY 350, Oxford Instruments plc; Oxford, UK) was employed to scrutinize the surface morphologies and ascertain the elemental composition of the materials, respectively. X-ray diffraction (XRD, D8 DISCOVER, Bruker AXS GmbH; Karlsruhe, Germany) analysis was conducted utilizing a Cu Kα radiation source at 40 kV and 30 mA, with scans ranging from 5° to 80° in 2θ. The surface composition and chemical states of the elements within the NFPC were elucidated using X-ray photoelectron spectroscopy (XPS; Thermo Scientific ESCALAB 250Xi, Thermo Fisher Scientific; Waltham, MA, USA). Fourier transform infrared spectroscopy (FTIR; Thermo Nicolet 6700, Thermo Fisher Scientific; Waltham, MA, USA) was applied to identify functional groups on the NFPC surface within the 4000 to 600 cm^−1^ wavenumber range. Additionally, the surface potential of a 100 mg·L^−1^ NFPC solution was quantified across a pH gradient of 3 to 9 using a Zetasizer nano instrument (Nano ZS90, Malvern Instruments Ltd.; Malvern, UK).

### 3.4. Adsorption and Desorption Experiments

We investigated the adsorptive performance of NFPC towards Sr^2+^ in aqueous solutions, meticulously evaluating a spectrum of pertinent experimental parameters. Preliminary adsorption studies were executed with a 0.4 g·L^−1^ NFPC solution, comprising 0.02 g of adsorbent in 50 mL of the target solution, against a backdrop of a 5 mg·L^−1^ Sr^2+^ solution. The influence of various parameters on the adsorption efficacy was appraised, including contact time (ranging from 10 to 220 min), solution pH (varied between 3 and 9), temperature (specifically at 303, 313, and 323 K), initial Sr^2+^ concentration (spanning 10 to 200 mg·L^−1^), and the presence of competing ions (K^+^, Na^+^, Ca^2+^, and Mg^2+^), all of which are critical in an engineering context. Equilibrium adsorption assays were uniformly performed on thermostatted shakers at 140 revolutions per minute. Post-experimental processing involved filtration of all samples through 0.22 μm pore-sized membranes, after which Sr^2+^ concentrations were quantified using an atomic absorption spectrophotometer (AAS; ContrAA700, Analytik Jena GmbH, Jena, Germany). Leaching experiments were conducted to investigate the potential leachability of strontium from Sr@NFPC under various durations (4 h, 8 h, and 12 h) and solution pH levels (ranging from 1 to 13 with increments of 2). Sr@NFPC was prepared by adsorbing NFPC in a 50 mL strontium solution with a concentration of 5 mg·L^−1^ at pH 7 for 120 min, followed by vacuum drying at 60 °C for 12 h. All experiments were performed on thermostatted shakers at 303 K with a rotation speed of 140 rpm. For reusability assays, Sr^2+^ was desorbed from NFPC via a 0.1 M HCl treatment for 4 h, followed by successive washing with deionized water until the supernatant’s pH approximated neutrality and Sr^2+^ could not be detected. The protocol for solid–liquid separation encompassed high-speed centrifugation. Adsorption, leaching, and desorption trials were replicated a minimum of two times to ensure the reliability of the findings. The adsorption performance parameters and models were depicted in Text S1 and Text S2, respectively.

### 3.5. DFT Calculation

Density functional theory (DFT) calculations delineate the electronic distribution, structural attributes, and interatomic interactions between Sr^2+^ and NFPC. The CP2K code [81] is an advanced computational tool that conducts ab initio molecular dynamics (AIMD) simulations across diverse states, encompassing solids, liquids, molecules, periodic systems, and crystals [82,83,84]. It is uniquely suited for modeling the interactions of periodic materials with charged particles, exemplified by the adsorption of Sr^2+^ onto NFPC. To comprehensively understand the adsorption mechanism, we harnessed the CP2K code, utilizing DFT to simulate and dissect the adsorption process meticulously. The detailed calculation method was described in Text S3.

## 4. Conclusions

The article systematically studied the adsorption performance and interaction mechanisms of NFPC for Sr^2+^ based on adsorption experiments, characterization analysis, and DFT calculation results for the first time. The adsorbent exhibited a broad pH applicability range (4–9), exceptionally high adsorption selectivity (with *K_d_* values surpassing 5000 mL·g^−1^ even in the presence of interfering ions at concentrations over 40 times higher than Sr^2+^), swift adsorption kinetics (equilibrium time was 36 min), substantial adsorption capacity (*Q* = 99.6 mg·g^−1^), and outstanding regenerative characteristics. Kinetics and isotherm analysis indicated that Sr^2+^ adsorption was a monolayer chemical process predominantly affected by film diffusion. Thermodynamic model analysis discloses that Sr^2+^ adsorption is an endothermic, entropy-increasing, spontaneous process. Combining experimental data with DFT-calculated configurations, we observed that Sr^2+^ participates in an ion exchange reaction with Na^+^ during adsorption and then coordinates with four oxygen atoms on the NFPC (100) surface. The PDOS results demonstrated the formation of Sr-O bonds through orbital hybridization between oxygen atoms and Sr^2+^ ions. The differential charge density and Bader charge transfer analyses confirmed the transfer of electrons from the oxygen atoms to Sr^2+^, leading to stable chemical adsorption. Overall, NFPC stands out for its straightforward fabrication and superior adsorption capabilities, suggesting promising applications in the selective uptake of Sr^2+^ from low-level radioactive wastewater.

## Data Availability

The data are contained within the article.

## Supplementary Materials

The following supporting information can be downloaded at https://www.mdpi.com/article/10.3390/molecules29122908/s1: Text S1. Adsorption performance parameters; Text S2. Adsorption model. Text S3. DFT calculation. Table S1. Partial kinetic parameters of the Sr^2+^ adsorption by NFPC; Table S2. Fitting parameters obtained by Boyd model and Weber–Morris models; Table S3. Fitting parameters obtained by adsorption isotherm models; Table S4. Fitting parameters obtained by adsorption thermodynamic models.
