# Supplementary material for: Selective Adsorption of Sr(II) from Aqueous Solution by Na3FePO4CO3: Experimental and DFT Studies
Source: Molecules. 2024 Jun 19;29(12):2908. doi: 10.3390/molecules29122908 (PMC11206743; doi:10.3390/molecules29122908)
Supplement: Supplementary file 1 [file molecules-29-02908-s001.zip › molecules-3036205-supplementary.pdf]

Selective adsorption of Sr(II) from aqueous solution by $Na_3FePO_4CO_3$: Experimental and DFT studies

Yudong Xie, Xiaowei Wang, Jinfeng Men *, Min Zhu, Chengqiang Liang, Hao Ding, Zhihui Du, Ping Bao, Zhilin Hu

College of Nuclear Science and Technology, Naval University of Engineering, Wuhan 430033, China; 15971428144@163.com (Y.X.); actwang@sina.com (X.W.)

* Correspondence: D23182702@nue.edu.cn (J.M.)

Text S1. Adsorption performance parameters

The adsorptive interaction between Sr^{2+} and NFPC was quantified by determining the adsorbate removal rates (R, %), distribution coefficients (K_d, mL·g^{-1}), and adsorption capacities at specific time intervals (Q_t, mg·g^{-1}) and equilibrium (Q_e, mg·g^{-1}).

$$R = \frac{C_0 - C_e}{C_0} \times 100 \tag{1}$$

$$K_d = \frac{(C_0 - C_e)V}{mC_e} \tag{2}$$

$$Q_t = \frac{(C_0 - C_t)V}{m} \tag{3}$$

$$Q_e = \frac{(C_0 - C_e)V}{m} \tag{4}$$

Where C_0 (mg·L^{-1}) and C_e (mg·L^{-1}) are the initial and equilibrium concentrations of adsorbates, respectively; V (mL) is the solution volume; m (mg) is the weight of adsorbent. Generally, a K_d value exceeding 5000 mL·g^{-1} indicates good adsorbate/adsorbent affinity; C_t (mg·L^{-1}) is the Sr^{2+} concentration after interaction with NFPC for a set duration.

Text S2. Adsorption model

S2.1 Adsorption kinetics models

We applied the Lagergren pseudo-first order [47], pseudo-second order [48], Elovich [49], Boyd [50], and Weber-Morris [51] models to decipher the adsorption mechanism to our experimental data.

Pseudo-first order model:

$$\ln(Q_e - Q_t) = \ln Q_e - K_1 t \tag{5}$$

Pseudo-second order model:

$$\frac{t}{Q_t} = \frac{1}{K_2 Q_e^2} + \frac{t}{Q_e} \tag{6}$$

$$h = K_2 Q_e^2 \tag{7}$$

$$t_{1/2} = \frac{1}{K_2 Q_e} \tag{8}$$

Elovich model:

$$Q_t = \frac{1}{\beta}\ln(\alpha\beta) + \frac{1}{\beta}\ln t \tag{9}$$

Where Q_e (mg·g^{-1}) and Q_t (mg·g^{-1}) are the adsorption capacity at equilibrium and time t (min), respectively; K_1 (min^{-1}) and K_2 (g·mg^{-1}·min^{-1}) are the pseudo-first-order and pseudo-second-order kinetic rate constant, respectively; h (mg·g^{-1}·min^{-1}) is the initial adsorption rate; $t_{1/2}$ (min) is the time to reach half of the adsorption equilibrium; α (mg·g^{-1}·min^{-1}) and β (mg·g^{-1}) are the initial elovich rate constant and desorption elovich rate constant, respectively.

Boyd model:

$$B_t = K_B t + I_B \tag{10}$$

$$B_t = -0.4977 - \ln(1 - \frac{Q_t}{Q_e}) \tag{11}$$

Weber-Morris model:

$$Q_t = I_W + K_W t^{1/2} \tag{12}$$

Where B_t is the function of Q_t/Q_e, K_B (min) and I_B are the constant and intercept of the Boyd model, respectively; K_W (mg·g^{-1}·min$^{-1/2}$) and I_W are the diffusion rate constant and intercept of the Weber-Morris model, respectively.

S2.2 Adsorption isotherm models

The adsorption isotherms were interpreted using the Langmuir [52,53], Freundlich [54], Dubinin-Radushkevich (D-R) [55], and Temkin [56] models, respectively:

Langmuir model:

$$\frac{C_e}{Q_e} = \frac{C_e}{Q_m} + \frac{1}{K_L Q_m} \tag{13}$$

$$R_L = \frac{1}{1 + K_L C_0} \tag{14}$$

Freundlich model:

$$\ln Q_e = \ln K_F + \frac{1}{n} \ln C_e \tag{15}$$

D-R model:

$$\ln Q_e = \ln Q_{DR} - K_{DR} \varepsilon^2 \tag{16}$$

$$\varepsilon = RT \ln(1 + \frac{1}{C_e}) \tag{17}$$

$$E = \frac{1}{\sqrt{2K_{DR}}} \tag{18}$$

Temkin model:

$$Q_e = \beta_T \ln K_T + \beta_T \ln C_e \tag{19}$$

Where C_e ($mg \cdot L^{-1}$) is the concentration at adsorption equilibrium; Q_m ($mg \cdot g^{-1}$) and Q_{DR} ($mg \cdot g^{-1}$) are the maximum adsorption capacity predicted by the Langmuir model and D-R model, respectively; K_L ($L \cdot mg^{-1}$), K_F ($mg^{1-1/n} \cdot g^{-1} \cdot L^{1/n}$), K_{DR} ($mol^2 \cdot kJ^{-2}$) and K_T ($kJ \cdot mol^{-1}$) are the equilibrium constant of Langmuir isotherm model, Freundlich isotherm model, D-R isotherm model and Temkin isotherm model, respectively; The separation coefficient (R_L, dimensionless) provides insight into adsorption favorability, with R_L values between 0 and 1 indicating favorable adsorption, and values above 1 suggesting unfavorable conditions; n is the Freundlich intensity parameter; ε ($J \cdot mol^{-1}$) is the Polanyi potential; R (8.314 $J \cdot mol^{-1} \cdot K^{-1}$) is the gas constant; T (K) is the Kelvin temperature; E ($kJ \cdot mol^{-1}$) is the mean adsorption energy, which distinguishes between chemisorption (E > 8) and physisorption (E < 8); β_T ($L \cdot mg^{-1}$) is the Temkin constant.

S2.3 Adsorption thermodynamics models

Thermodynamic parameters, including the Gibbs free energy change (ΔG^0, $kJ \cdot mol^{-1}$), entropy change (ΔS^0, $J \cdot mol^{-1} \cdot K^{-1}$), and enthalpy change ($\Delta H^0$, $kJ \cdot mol^{-1}$), were calculated to understand temperature's impact on the adsorption process [57].

$$\ln K_d^0 = \frac{\Delta S^0}{R} - \frac{\Delta H^0}{RT} \tag{20}$$

$$\Delta G^0 = \Delta H^0 - T\Delta S^0 \tag{21}$$

$$K_d^0 = K_d \times \frac{C^0}{Q^0} \tag{22}$$

Where R (8.314 $J \cdot mol^{-1} \cdot K^{-1}$) is the gas constant; T (K) is the Kelvin temperature; K_d^0 is the dimensionless equilibrium constant; C^0 and Q^0 are the standard states of solution in liquid and solid, respectively. According to IUPAC regulations, C^0 is 1 $mol \cdot L^{-1}$. When the adsorbate is not a gas, we suggest Q^0 is set to1 $mol \cdot kg^{-1}$.

Text S3. DFT calculation

Our DFT calculations utilized the CP2K code, employing a hybrid Gaussian and plane-wave basis set approach. Core electrons were modeled using the norm-conserving Goedecker-Teter-Hutter pseudopotentials. The valence electron wavefunctions were expanded in a double-zeta basis set augmented with polarization functions, further complemented by an auxiliary plane-wave basis set with an energy cutoff threshold set at 400 eV. The generalized gradient approximation exchange-correlation functional by Perdew, Burke, and Ernzerhof (PBE) was selected for our simulations. Each configuration was optimized using the Broyden-Fletcher-Goldfarb-Shanno (BFGS) algorithm, with self-consistent field (SCF) convergence criteria set to 1.0×10^{-6} au. To account for the long-range van der Waals dispersion interactions, we integrated the DFT-D3 scheme, with an empirical dispersion correction, into the energies derived from the exchange-correlation functional. Furthermore, the self-consistent continuum solvation (SCCS) model, calibrated with a dielectric constant of 78.54, was engaged to replicate the aqueous solvent environment pertinent to the Sr^{2+} adsorption process.

The binding energy(BE) between NFPC and the Sr^{2+} ion was computed employing the following equation:

$$BE = E_{\mathrm{Sr^{2+}/NFPC}} - E_{\mathrm{NFPC}} - E_{\mathrm{Sr^{2+}}} \qquad (22)$$

Where $E_{\mathrm{Sr^{2+}/NFPC}}$ and E_{NFPC} represent the total energies of the NFPC with and without the adsorption of the Sr^{2+} ion, respectively. $E_{\mathrm{Sr^{2+}}}$ is the total energy of the Sr^{2+} ion. As this equation dictates, a negative BE indicates a stable adsorption complex.

Table S1 Partial kinetic parameters of the Sr^{2+} adsorption by NFPC.

Adsorbate	Pseudo-first-order model		Pseudo-second-order model				Elovich model	
	Q_e (mg·g^{-1})	K_1 (min^{-1})	Q_e (mg·g^{-1})	K_2 (g·mg^{-1}·min^{-1})	H (mg·g^{-1}·min^{-1})	$t_{1/2}$ (min)	A (mg·g^{-1}·min^{-1})	β (g·mg^{-1})
Sr^{2+}	1.94	2.70E-2	12.58	4.41E-3	0.67	18.04	475.99	1.02

Table S2 Fitting parameters obtained by Boyd model and Weber-Morris models.

Adsorbate	Boyd model		Weber-Morris model	
	K_B (min^{-1})	I_B	K_W (mg·g^{-1}·min$^{-0.5}$)	I_W (mg·g^{-1})
Sr^{2+}	6.73E-2	0.13	4.41E-2	11.66

Table S3 Fitting parameters obtained by adsorption isotherm models.

Adsorbate	Langmuir		Freundlich		D-R			Temkin	
	Q_m (mg·g^{-1})	K_L (L·mg^{-1})	K_F ((mg·g^{-1})·(L·mg^{-1})$^{1/n}$)	N --	Q_{DR} (mg·g^{-1})	K_{DR} (mol^2·KJ^{-2})	E (KJ·mol^{-1})	β_T (L·mg^{-1})	K_T (KJ·mol^{-1})
Sr^{2+}	63.48	0.16	24.97	6.82	2.61	1.99E-3	15.82	8.65	11.86

Table S4 Fitting parameters obtained by adsorption thermodynamic models.

Adsorbent/Adsorbate	T (K)	K_d^0	ΔH^0 (kJ·mol^{-1})	ΔS^0 (J·mol^{-1}·K^{-1})	ΔG^0 (kJ·mol^{-1})
NFPC /Sr^{2+}	303	1.01	-121.38	-39.92	-109.28
	313	0.87			-108.89
	323	0.75			-108.49
